# Leveraging the transcriptome-phenotype relationship to guide clinical management of papillary thyroid cancer

**DOI:** 10.3389/fendo.2026.1737469

**Published:** 2026-01-28

**Authors:** Adrian Harvey, Eric Walser, Rebecca Lahamm-Andraos, Caitlin Yeo, Samantha Wolfe, Cynthia Stretch, Steven Craig, Oliver F. Bathe

**Affiliations:** 1 Department of Surgery, Cumming School of Medicine, University of Calgary, Calgary, AB, Canada; 2 Department of Surgery, Schulich School of Medicine & Dentistry, Western University, London, ON, Canada; 3 Department of Surgery, West Virginia University, Morgantown, WV, United States; 4 Department of Oncology, Cumming School of Medicine, University of Calgary, Calgary, AB, Canada; 5 Qualisure Diagnostics, Calgary, AB, Canada; 6 Graduate School of Medicine, University of Wollongong, Wollongong, NSW, Australia; 7 Arnie Charbonneau Cancer Institute, University of Calgary, Calgary, AB, Canada

**Keywords:** gene-expression classifier, molecular diagnostics, papillary thyroid carcinoma, precision oncology, radioactive iodine, risk stratification, Thyroid GuidePx^®^, transcriptomics

## Abstract

**Background:**

Papillary thyroid carcinoma (PTC) is the most common endocrine malignancy, with excellent survival but substantial variation in recurrence risk. Traditional clinicopathologic risk models, while still a cornerstone of current guidelines, overlook the biological differences between patients, resulting in both overtreatment and undertreatment.

**Content:**

Next-generation sequencing has advanced our molecular understanding of PTC by identifying recurrent driver mutations that shed light on tumor initiation. However, mutations fall short of explaining the full spectrum of clinical behavior. DNA-based mutation profiling offers a fixed snapshot of genetic alterations, while transcriptomics captures the tumor’s active biological state, integrating signaling pathways, differentiation status, immune interactions, and metabolism. Large-scale efforts like The Cancer Genome Atlas, along with emerging transcriptomic classifiers, have shown that gene-expression subtypes (“BRAF-like” and “RAS-like”) more accurately predict iodine avidity, tumor aggressiveness, and treatment response than histology or genotype alone. Transcriptome-based tools such as Thyroid GuidePx^®^ now allow for biologically informed risk stratification that goes beyond traditional clinicopathologic and mutation-only approaches.

**Summary and outlook:**

In the preoperative setting, transcriptomic testing can inform whether patients are best suited for active surveillance, lobectomy, or total thyroidectomy. Postoperatively, it sharpens decisions around completion surgery, radioactive iodine use, and the intensity of TSH suppression. Integrating transcriptomic data into clinical decision-making enables more precise selection for treatment escalation or de-escalation. To unlock the full potential of transcriptome-guided management in PTC, prospective validation and adoption into ATA and NCCN guidelines will be critical.

## Introduction

The incidence of differentiated thyroid cancer is rising; it is now the 7th most common cancer worldwide, with >800,000 new cases annually (1). Most cases are low risk with excellent outcomes. In the U.S., all-cause mortality for low-risk disease is <1% (2) and recurrence is <5% (3). Given the expectation of long-term survival, attention has shifted to avoiding overtreatment and using healthcare resources judiciously. De-escalating care in appropriately selected patients can reduce treatment-related morbidity, unnecessary resource use, and patient anxiety.

There are multiple key decision points in the management and follow-up of differentiated thyroid cancer. These include a) active surveillance vs surgery for small unifocal cancers with no lymph node involvement; b) extent of surgery; c) postoperative use of radioactive iodine (RAI); and d) surveillance intensity (**Table 1**). Optimal choices at each step in the care pathway depend heavily on the ability to accurately estimate prognosis in individual patients. Historically, risk estimation has relied on clinicopathologic features. The 2015 ATA guidelines stratified patients into low, intermediate, and high risk, with management recommendations according to this risk (3). The 2025 update refines this into low, low-intermediate, high-intermediate, and high risk (4). However, clinicopathologic systems explain only part of outcome variability and can lead to over- or undertreatment. Examples of this disconnect are well documented (5, 6). Active surveillance spares many patients with small, low-risk cancers from surgery, yet a minority demonstrate growth or nodal metastasis requiring delayed operation (7, 8). Similarly, although routine prophylactic central neck dissection often reveals occult nodal disease in >1/3 of patients, recurrence rates are comparable to cohorts managed without prophylactic dissection (9–11). These and other observations highlight the limits of traditional risk stratification systems: while useful for population-level guidance, they lack granularity for truly personalized care. Notably, a structurally incomplete response to initial therapy, while more common in higher risk categories, is still observed in up to 5% of low-risk patients and only ~33% of high-risk patients without distant metastases (12–15).

**Table 1:** Key decision points in the clinical management pathway of PTC.

Phase of care	Decision point	Traditional basis for decision	Limitations of conventional approach	Potential role for molecular / transcriptomic data
Diagnosis	Evaluation of thyroid nodule (surgery vs observation)	Ultrasound features, cytology (Bethesda system)	Indeterminate cytology (Bethesda III/IV) leads to diagnostic surgery in many benign nodules	Molecular classifiers (e.g., Afirma®, ThyroSeq®) refine benign vs malignant risk and reduce unnecessary surgery
Preoperative Management	Active surveillance vs. surgery or ablation for microcarcinoma (<1 cm)	Tumor size, multifocality, clinical context	Some small PTCs progress; others remain indolent	Gene-expression signatures and mutational data may identify biologically aggressive small tumors
	Extent of initial surgery (lobectomy vs total thyroidectomy)	Tumor size, multifocality, extrathyroidal extension	Pathologic risk factors often unknown preoperatively; 11–34% need completion thyroidectomy	Preoperative transcriptomic risk classifiers (e.g., Thyroid GuidePx®) may predict need for total thyroidectomy and may more confidently identify candidates for de-escalation
	Prophylactic central neck dissection	Clinical node status, surgeon preference	Occult nodal metastases common but rarely alter outcomes	Transcriptomic markers of invasion or EMT may refine patient selection
Immediate Postoperative Management	Need for completion thyroidectomy	Postoperative histopathology	Subjective interpretation of vascular invasion and variant morphology	Transcriptomic signatures may indicate biological risk more accurately than morphology alone
	Radioactive iodine (RAI) ablation: indication and dose	ATA risk category, tumor size, nodal status	Intermediate-risk category is heterogeneous; RAI benefit uncertain in many	Transcriptomic signatures of iodine-handling and differentiation may predict RAI sensitivity and may guide dosing
	TSH suppression intensity and duration	ATA risk category, response to therapy	Overtreatment increases risk of atrial fibrillation and osteoporosis; undertreatment may miss recurrence risk	Integration of transcriptomic risk profiles may tailor TSH targets to biological aggressiveness
Post-treatment Surveillance	Frequency and intensity of follow-up	ATA response-to-therapy classification	Morphologic and biochemical criteria may not fully reflect recurrence risk	Molecular risk models could inform personalized surveillance intervals
Recurrent or Persistent Disease	Management of locoregional recurrence	Anatomic extent, previous therapy	Variable natural history; risk of overtreatment	Transcriptomic classifiers may guide reoperation vs. surveillance
	Management of distant metastases / RAI-refractory disease	Imaging, RAI uptake, thyroglobulin trend	RAI refractoriness difficult to predict; systemic therapy selection empirical	Transcriptomic and genomic profiling identify candidates for redifferentiation therapy, TKIs, or immunotherapy
Advanced / Systemic Therapy	Selection of targeted therapy	NGS-defined mutations/fusions (BRAF, RET, NTRK, ALK, etc.)	Not all mutations confer equal drug sensitivity	Actionable mutations currently guide drug selection. Integration of transcriptomic data may refine treatment selection.

Molecular profiling has *potential* to inform nearly every decision point along the PTC care pathway. In particular, transcriptome-based tests provide biologically grounded risk information that complements clinicopathologic assessment and enables more personalized, evidence-based management.

More precise prognostication should explain a greater share of outcome variance. Ideally, prognostication should be available early in the care pathway, where its impact is maximal. For instance, lobectomy is often chosen for localized cancers <4 cm to avoid overtreatment with total thyroidectomy, yet 20–50% subsequently require completion thyroidectomy (16–18), with a systematic review estimating 11–34% (19). This is typically due to higher-risk pathology identified only postoperatively. Completion thyroidectomy places patients at risk related to surgery, including hypothyroidism, hypoparathyroidism, and decreased quality of life, while also increasing health care burden (20, 21).

**Figure 1:**
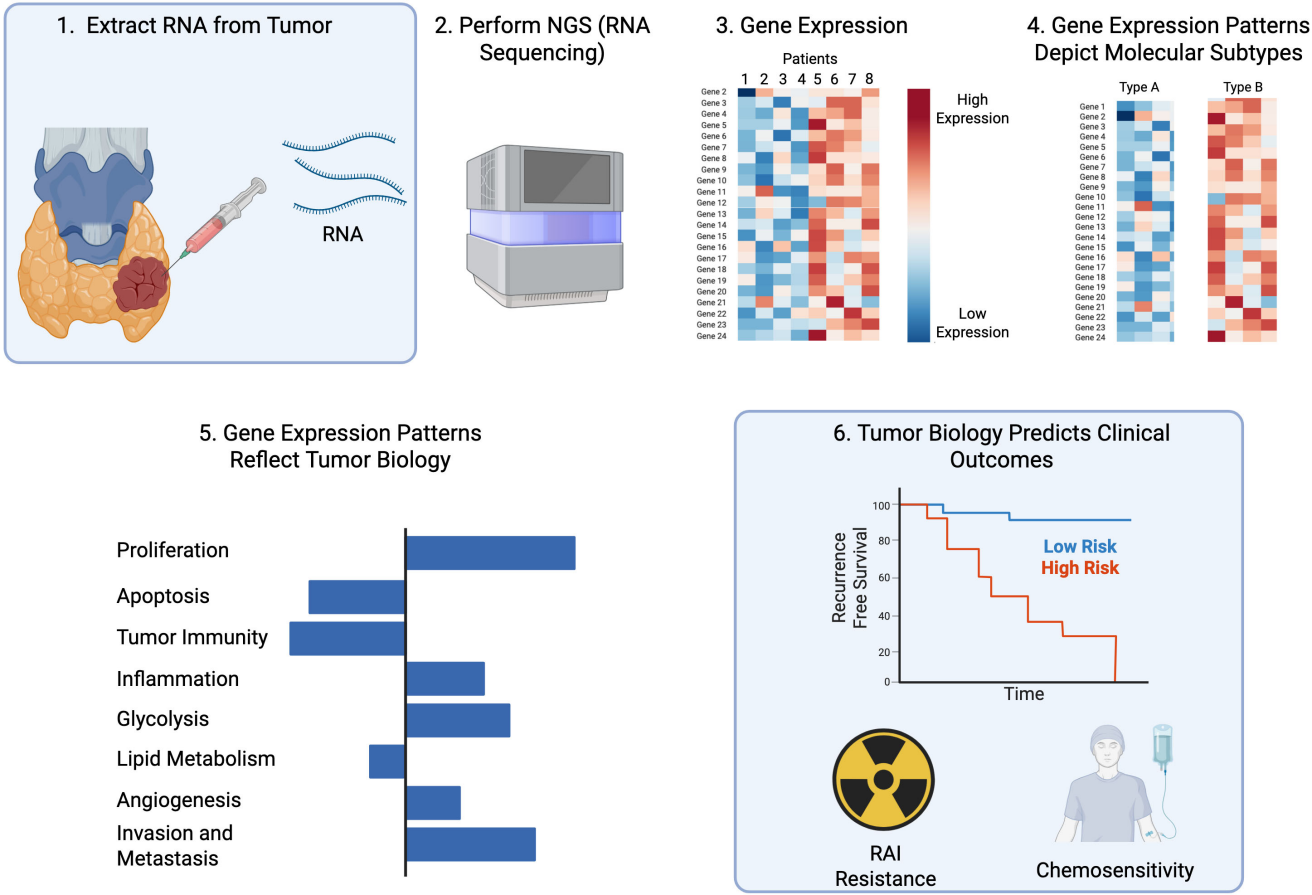
Transcriptomic profiling enables biologic risk stratification. Gene expression profiling requires only a small amount of tissue to enable next generation sequencing (NGS; RNASeq). The pattern of gene expression facilitates molecular subtyping and detailed analysis of biological features. The biological features of a cancer are highly predictive of clinical outcomes, including recurrence risk, sensitivity to RAI, and drug sensitivity. Created with Biorender.com.

The advent of next-generation sequencing (NGS) has transformed the molecular characterization of thyroid lesions, allowing analysis of genomic and transcriptomic features, separately and in parallel. NGS-based approaches not only identify oncogenic drivers but also capture the transcriptomic programs that more closely reflect tumor phenotype (**Figure 1**). This forms the foundation for precision prognostication and biologically guided management of papillary thyroid cancer (PTC).

Molecular analysis, first adopted to refine malignancy risk in indeterminate fine-needle aspiration (FNA) cytology, now shows promise for improving risk stratification in confirmed cancer. Both mutational profiling (DNA) and interrogation of the transcriptome (mRNA) can augment clinicopathologic models to better tailor treatment and surveillance. If reliably obtained from FNA, these metrics could influence decisions earlier in the treatment pathway.

In this review, we explore how the evolving understanding of the transcriptome–phenotype relationship can inform individualized management of PTC, spanning preoperative, postoperative, and emerging therapeutic decision points.

## The molecular landscape of papillary thyroid cancer

The molecular landscape of PTC has been defined over decades of research, culminating in a detailed map of driver mutations and co-related features annotated by The Cancer Genome Atlas (22, 23). These efforts have demonstrated that most PTCs are driven by MAPK-pathway activation via *BRAF^V600E^* or *RAS* mutations (22, 24); a minority are driven by receptor tyrosine kinase fusions including *RET* and *NTRK* fusions (25). These alterations shape phenotype, iodine avidity, and (with co-mutations) prognosis, but they incompletely explain outcome variability.

The *BRAF* gene encodes a key intracellular signaling protein in the *MAPK*/*ERK* pathway, regulating cellular proliferation. The *BRAF^V600E^* mutation is common, present in 29-83% of PTC. Its presence can initiate tumorigenesis in normal thyroid follicular cells, increasing cellular proliferation, inhibiting apoptosis, and encouraging de-differentiation (25). By itself, the presence of the *BRAF^V600E^* mutation is not prognostic (26–29). However, prognostic value improves when considered with co-mutations (e.g., *BRAF^V600^* + *TERT*, *BRAF^V600^* + *RAS*) (30, 31) and the transcriptomic state (26). Therapeutically, *BRAF^V600E^* provides a target for systemic therapy in advanced disease (32, 33).

The three *RAS* isoforms (*HRAS*, *KRAS*, and *NRAS*) are small GTPases involved in signal transduction in the MAPK and PI3K-AKT pathways. Mutations result in constitutive activation of the MAPK and PI3K-AKT pathways, driving proliferation and growth, as well as promoting cell survival. *NRAS* mutations account for the majority of RAS alterations, followed by *HRAS*, with *KRAS* being least frequent. *RAS*- mutated PTCs tend to demonstrate follicular morphology, often behaving more like follicular thyroid cancer. Generally, *RAS*-mutated PTCs have higher iodine avidity and less aggressive behavior than *BRAF^V600E^* mutated PTCs, consistent with feedback-attenuated MAPK signaling (22, 34, 35).

The *TERT* promoter controls transcription of the telomerase reverse transcriptase gene. Point mutations at positions –124 (C228T) and –146 (C250T) lead to upregulation of TERT transcription, leading to increased telomerase activity and maintenance of telomere length, enabling replicative immortality (36). *TERT* mutations are associated with aggressive forms of thyroid cancer, occurring commonly in anaplastic and poorly differentiated thyroid cancer (37, 38). Although uncommon in PTC (~10% of PTC specimens), it is consistently linked to worse outcomes, especially when coupled with *BRAF* or *RAS* mutations. Co-mutation status refines recurrence risk beyond single-gene calls (30, 37, 39).

The *RET* gene encodes a transmembrane receptor tyrosine kinase that normally regulates cell growth and differentiation, particularly in neural crest–derived and renal tissues. In PTC, *RET* is activated through gene fusions (most commonly *CCDC6-RET* and *NCOA4-RET*), which drive constitutive kinase activation and downstream MAPK signaling (40). *RET* fusions are found in ~5–10% in adult PTC, and more frequently in pediatric or radiation-associated PTC (41). RET is not normally expressed in thyroid follicular cells, so its activation via fusion is oncogenic and therapeutically targetable (42, 43).

*NTRK* refers to three homologous genes: *NTRK1*, *NTRK2*, and *NTRK3*, encoding the neurotrophic tyrosine kinases TRKA, TRKB and TRKC, respectively. In PTC, the most common *NTRK* fusions are *ETV6-NTRK3*, *TPM3-NTRK1*, and *TPR-NTRK1* (33, 41, 44, 45). They occur in ~2–3% of adult PTCs but are more common in radiation-induced and pediatric cases. Functionally, NTRK fusions result in persistent activation of MAPK and PI3K–AKT signaling, driving uncontrolled proliferation, de-differentiation, and survival. Phenotypically, tumors often show solid or follicular architecture and higher iodine avidity than BRAF-like cancers. NTRK fusions are highly actionable, with dramatic and durable responses to TRK inhibitors such as larotrectinib and entrectinib (46–48).

A number of low frequency mutations and fusions have been reported in PTC. Their rarity limits stand-alone prognostic utility. However, they may add context when integrated with transcriptomic state and clinicopathology (49–51).

The presence of *TP53* mutations is associated with aggressive behavior when found in PTC, and has been associated with progression to anaplastic thyroid cancer (52). *EIF1AX* mutations are found rarely (1-2.5%) in PTC, frequently co-existing with RAS mutations. They can also be found in benign lesions (50). The largest series reported is only 31 cases, and it was not possible to definitively describe outcomes that associated with *EIF1AX* mutations (53). Aberrant activation of the PI3K–AKT–mTOR pathway is known to promote cell proliferation, survival, and progression; PI3K and MAPK pathways frequently co-activate and cross-talk in advanced disease. *PIK3CA* mutations are more common to follicular and aggressive forms of thyroid cancer (49). *PAX8-PPARG*, a fusion of the *PAX8* transcription factor and the *PPARG* adipogenesis regulator, is commonly identified in follicular adenomas, noninvasive follicular thyroid neoplasm with papillary-like nuclear features as well as follicular variant of PTC. When present in invasive thyroid cancer there are mixed reports on *PAX8-PPARG* on a marker of aggressiveness (54–56). *ALK* fusions (most commonly with *STRN* or *EML4* in thyroid cancer) are identified in 1-3% of PTCs. There is some evidence suggesting aggressive behavior when *ALK* fusions are found in PTC (41, 51, 57).

**Figure 2:**
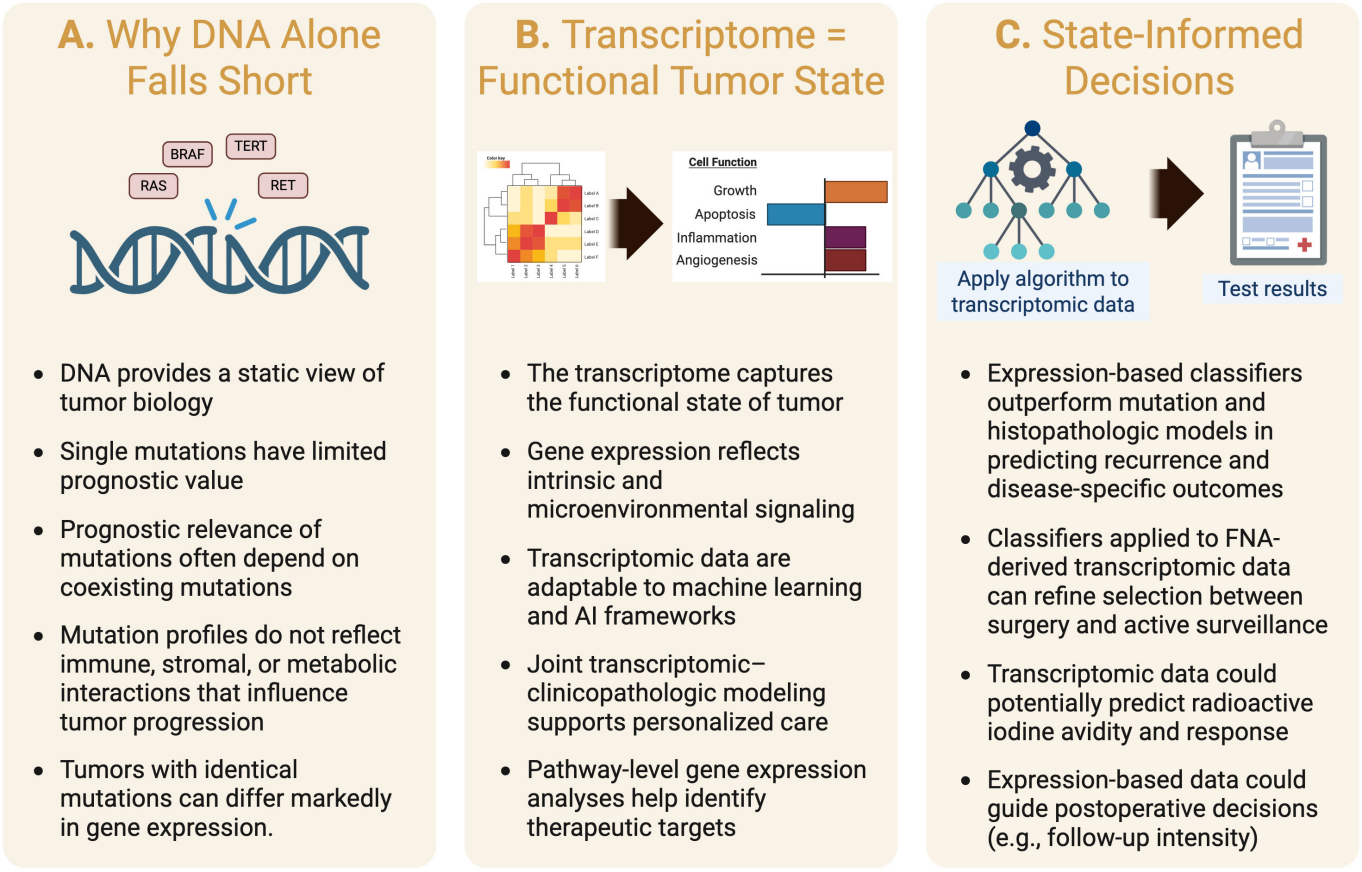
From signals to choices: transcriptome-defined tumor state can guide management in papillary thyroid cancer. **(A)** Why DNA alone is limited. Mutations/fusion profiling (e.g., *BRAF*, *RAS*, *TERT*, *RET/NTRK*) provides a static snapshot and does not fully reflect tumor behavior. The prognostic impact often depends on co-existent alterations, and mutation profiles do not capture immune, stromal, or metabolic interactions. **(B)** Transcriptome = functional tumor state. Gene-expression programs integrate tumor-intrinsic and microenvironmental signals reflected in the phenotype. High-dimensional transcriptomic data lend themselves to predictive modeling, enabling the development of robust, scalable, and continuously improving diagnostic tools. Furthermore, integrating clinicopathologic information with transcriptomic data can further individualize treatment. **(C)** State-informed decisions. Classifiers applied to transcriptomic data could enhance treatment preoperatively and postoperatively. FNA-derived transcriptomic data could refine preoperative risk assessments, refine selection for surgery versus active surveillance, and determine the appropriate extent of resection. Postoperatively, expression-based models complement histopathology to inform completion thyroidectomy, RAI decisions, and follow-up intensity. Created with Biorender.com.

Collectively, these driver events provide a foundational understanding of oncogenic initiation in thyroid cancer and have informed both preoperative diagnostic testing and targeted therapies for advanced disease. Yet, despite this detailed genomic map, mutation profiles alone fail to fully account for the biological and clinical heterogeneity of PTC, as explored in the next section (**Figure 2**).

## Limitations of mutation-focused approaches

Although genomic profiling has illuminated the recurrent driver events in PTC, its prognostic and predictive value is inherently limited. Most mutations represent *initiating events* in tumorigenesis but do not capture the downstream regulatory processes that determine phenotype, therapeutic sensitivity, or clinical outcome. For example, *BRAF^V600E^*, while common, has inconsistent correlations with recurrence, and its high prevalence (~50%) reduces its discriminative utility. Even *TERT* promoter mutations, which more consistently associate with aggressive behavior, exert variable effects depending on co-mutational context. Low-frequency mutations such as *TP53*, *EIF1AX*, or *PIK3CA*, though mechanistically interesting, occur too rarely to inform population-level risk stratification.

Importantly, DNA-based testing provides a static snapshot of potential, not a dynamic measure of function (**Figure 2**). Mutations identify pathway activation capacity but not its actual transcriptional or phenotypic expression. They fail to reflect the contributions of epigenetic regulation, microRNA networks, metabolic reprogramming, and immune–stromal interactions, all of which shape tumor behavior. Intratumoral heterogeneity further complicates interpretation, as variant allele frequency can vary across tumor regions, influencing MAPK output and iodine avidity. Moreover, mutation burden does not necessarily predict signaling intensity or treatment response. For example, although *BRAF^V600E^* mutation is commonly associated with reduced radioactive iodine (RAI) avidity, the correlation is far from perfect: a subset of *BRAF^V600^*^E^-mutant tumors maintain iodine uptake (58, 59).

Collectively, these limitations illustrate that mutation profiling defines the *genetic architecture* of a tumor but not its *functional state* or biological behavior. The clinical phenotype (growth rate, differentiation, immune milieu, and therapeutic response) emerges from the integration of genetic, epigenetic, and microenvironmental factors operating at the transcriptional level. Recognizing this gap has shifted the field toward a transcriptome-based framework, where gene expression patterns reflect the realized state of the tumor rather than its latent potential. This transition from identifying oncogenic drivers to understanding the functional programs they activate marks a critical inflection point in thyroid cancer biology and underpins the emerging emphasis on the transcriptome as the key to precision prognostication.

## The transcriptome-phenotype relationship in PTC

The transcriptome–phenotype relationship provides a more accurate and comprehensive reflection of PTC biology than conventional histopathological classification or mutational profiling. Large-scale integrative studies such as TCGA have shown that gene expression–based subtypes (“*BRAF*-like” and **“***RAS*-like”) better predict tumor differentiation, immune microenvironment, and therapeutic response than histologic variants (23). Gene set enrichment analysis (GSEA) further extends these insights by identifying coordinated changes across predefined groups of genes representing biological pathways or cellular functions (**Figures 1**, **2**). Indeed, this approach was used to derive a clinically useful and biologically informative transcriptomic classifier for PTC, demonstrating that enrichment of MAPK, PI3K/AKT, metabolic, and immune signaling programs distinguishes molecular subtypes and accounts for differences in tumor differentiation, iodine avidity, and clinical behavior (26).

Single-cell transcriptomic analyses have expanded this framework, revealing distinct malignant thyrocyte phenotypes: follicular-like, partial EMT-like, and dedifferentiation-like (60). Spatial transcriptomics has also facilitated the identification of important ligand-receptor interactions (e.g., FN1–SDC4, CXCL13–CXCR5) that may be important in the transition of follicular cells to PTC cells (61). While these single-cell and spatial approaches provide invaluable mechanistic insight, they are not yet applicable in routine clinical practice. In contrast, bulk transcriptomic profiling has now reached clinical translation, with validated assays demonstrating prognostic and predictive value in guiding management of PTC (26).

Beyond mechanistic insights, transcriptomic profiling holds significant clinical utility in prognostication and treatment stratification. Gene-based assays such as ThyroSeq^®^ and Afirma^®^ have already transformed the management of indeterminate thyroid nodules by reducing unnecessary surgeries (3, 62–64). These molecular tests are fundamentally distinct in design and biological focus. For example, ThyroSeq^®^ is a mutation- and fusion-based panel that identifies oncogenic drivers to refine diagnostic certainty (“rule in” malignancy), whereas Afirma^®^ is a transcriptome-based expression classifier that differentiates benign from malignant nodules based on global gene-expression patterns (“rule out” malignancy). Both were developed for diagnostic triage in indeterminate nodules, not for prognostication once PTC is confirmed.

Newer transcriptome-derived approaches now extend molecular testing beyond diagnosis to capture biological behavior and recurrence risk (26, 65, 66), marking a conceptual shift from identifying malignancy to understanding tumor aggressiveness. Transcriptome-based prognostic classifiers are now being translated into clinical use. For example, Thyroid GuidePx^®^ is a validated multigene expression classifier developed to predict recurrence risk in PTC (26, 65). Unlike mutation-based panels, which primarily capture oncogenic initiation events, transcriptomic classifiers such as Thyroid GuidePx^®^ reflect the integrated biological state of the tumor, encompassing both tumor-intrinsic and microenvironmental signals. In validation studies, this approach demonstrated improved specificity for identifying indolent disease compared with ATA risk stratification, supporting its potential role in reducing overtreatment and informing individualized management pathways.

These insights set the stage for applying transcriptome-based classifiers to guide clinical decisions across the treatment pathway.

## Clinical utility of molecular testing

### Preoperative molecular testing

Molecular testing can be performed either preoperatively on FNA cytology or postoperatively on resected tumor tissue. While both approaches provide valuable biological information, their clinical utility differs substantially. Preoperative molecular testing offers the greatest opportunity to influence the entire treatment pathway, particularly in patients with early-stage PTCs measuring 1–4 cm, clinically negative lymph nodes, and who are candidates for thyroid lobectomy. In this setting, molecular results could guide the choice between active surveillance and surgery, determine the extent of resection, and anticipate the need for adjuvant radioactive iodine (RAI). Potentially, results could also inform decisions on nonsurgical ablation.

Initially, molecular testing was developed to improve diagnostic accuracy for indeterminate nodules (Bethesda III and IV), reducing unnecessary surgery. Until recently, its application in clearly malignant or suspicious nodules (Bethesda V and VI) was limited and largely investigational (67, 68). Mutation-based panels such as ThyroSeq^®^ can identify oncogenic drivers but are not designed to predict clinical behavior or long-term outcomes once PTC is diagnosed. In the experience reported by Schumm et al., for example, a large proportion of cancers were categorized as molecular intermediate risk, a broad and heterogeneous group that provides little actionable information for determining surgical extent or postoperative management (69). Thus, while these assays can complement clinicopathologic evaluation in select scenarios, they have limited value in guiding individualized treatment for patients with confirmed PTC.

Certain genetic alterations, when detected preoperatively, can still inform management decisions in selected cases. *TERT* promoter mutations, for example, are consistently associated with aggressive disease and poorer outcomes, and may support consideration of total thyroidectomy and postoperative RAI (70, 71), especially with concurrent *BRAF^V600E^* or *RAS* mutations (30, 37, 39). Conversely, isolated *RAS* mutations are typically found in encapsulated follicular variant PTC or non-invasive follicular thyroid neoplasm with papillary-like nuclear features (NIFTP) and generally predict indolent behavior, for which lobectomy alone is often sufficient (72). However, such genotype–phenotype correlations are incomplete and insufficiently precise for individualized management, particularly in tumors that fall between clear “low” and “high” risk boundaries. For this reason, the 2025 ATA guidelines do not recommend routine mutational testing to inform preoperative planning (Recommendation 10) (4).

Nevertheless, molecular testing from FNA specimens represents a powerful opportunity to integrate biologic insight earlier in the decision-making process before definitive surgery. In this context, transcriptomic profiling is emerging as a more informative approach than mutation testing, capturing the *functional state* of the tumor rather than its static genetic architecture.

Bulk RNA–based classifiers can be performed on preoperative material and have demonstrated superior accuracy over clinicopathologic or mutation-based models in predicting recurrence and aggressiveness (26). These assays integrate tumor-intrinsic and microenvironmental signals, offering a quantitative, biology-based framework for surgical and adjuvant planning. When applied preoperatively, transcriptomic classifiers have the potential to influence the entire treatment pathway, helping identify candidates for active surveillance, determining the optimal extent of surgery, and anticipating RAI sensitivity or resistance. As evidence accumulates, transcriptome-guided decision-making is poised to become a cornerstone of precision management in early-stage PTC.

### Postoperative molecular testing

Postoperative molecular testing refines prognostication to guide adjuvant therapy decisions, including the need for completion thyroidectomy and radioactive iodine (RAI) administration. Traditionally, postoperative risk assessment has relied on histopathologic features such as vascular invasion, capsular penetration, and variant histology. However, these parameters suffer from imperfect inter-observer concordance among pathologists (73–78). The association of vascular invasion and recurrence risk is not universally observed (79, 80), and vascular invasion may vary by tumor regions (73). The prognostic value of rare “aggressive” PTC variants such as hobnail variants is far from certain owing to small sample sizes. The proportion of tall cells that confers risk is unclear, and there is often disagreement between expert pathologists on what is or is not a tall cell (81, 82). These limitations contribute to substantial heterogeneity in outcomes among patients within the same ATA risk category, particularly in the intermediate-risk group. This variability underscores the need for biologically grounded markers that complement morphologic assessment.

Genomic profiling of resected tumors has provided insights into the mechanisms underlying disease progression. The coexistence of *TERT* promoter mutations with *BRAF^V600E^* or *RAS* mutations identifies tumors with increased risk of recurrence and mortality (83–86). However, tumors with the same genotype can show markedly different behavior, RAI avidity, and immune profiles. Reflecting these uncertainties, the 2025 ATA guidelines no longer make genotype-based recommendations for completion thyroidectomy, emphasizing instead the combined effect of multiple molecular and clinicopathologic factors (4).

The discordance often seen in mutational profiling points to the need for functional assays that reflect pathway activation, differentiation, and microenvironmental context. Transcriptomic profiling meets this need by measuring the aggregate expression of thousands of genes that together define tumor differentiation, metabolic state, and immune milieu. Bulk RNA-seq studies demonstrate that dedifferentiation signatures, immune suppression, and metabolic reprogramming correlate strongly with RAI refractoriness and recurrence (26, 66).

Building on these principles, Thyroid GuidePx^®^ was developed as a transcriptome-derived prognostic classifier specifically for PTC (26, 65). By integrating gene-expression patterns that capture tumor-intrinsic and microenvironmental biology, Thyroid GuidePx^®^ distinguishes indolent from aggressive disease beyond ATA risk categories. In validation cohorts, the assay demonstrated higher specificity for identifying low-risk tumors compared with clinicopathologic or mutation-based models, offering an opportunity to reduce the completion thyroidectomy rate, reduce unnecessary RAI, and extend surveillance intervals for biologically indolent cases. Its application to postoperative tissue provides a complementary layer of risk stratification that links molecular phenotype to therapeutic decision-making.

Another key postoperative decision, often underappreciated in discussions of individualized management, is the intensity and duration of thyroid-stimulating hormone (TSH) suppression. TSH suppression has long been used as an adjunct to surgery and RAI to reduce recurrence risk, based on the trophic effects of TSH on thyrocyte proliferation. However, evidence summarized in the 2025 ATA Guidelines (4) indicates that the intensity of suppression should be carefully tailored to recurrence risk and treatment response. For most low- and low-intermediate–risk patients with an excellent or indeterminate response, maintaining TSH within or just below the reference range (0.5–2 mIU/L) is now considered sufficient. In contrast, patients with persistent or structurally incomplete disease may benefit from more stringent suppression (<0.1 mIU/L), provided the benefits outweigh potential harms. Excessive or prolonged suppression can lead to iatrogenic subclinical thyrotoxicosis, increasing the risk of atrial fibrillation, left ventricular hypertrophy, and accelerated bone loss, particularly in postmenopausal women and older adults. These risks highlight the need for individualized titration based on biological risk rather than uniform suppression. Transcriptomic classifiers that better resolve tumor aggressiveness may ultimately support more nuanced TSH targets, avoiding overtreatment in biologically indolent disease while maintaining protective suppression in high-risk molecular phenotypes.

Decisions related to RAI are mostly based on recurrence risk and stage, but insufficiently grounded in science (87). The ATA recommends that patients with intermediate-low and intermediate-high risk DCT be considered for treatment with 30-100mCi; those at high risk of recurrence should receive 100-150mCi; and metastatic disease justifies higher doses of 100–200 mCi (4). A biomarker that reflects sensitivity to RAI is needed. It is known that *BRAF*-like tumors frequently demonstrate impaired iodine uptake due to suppression of the sodium-iodide symporter (NIS) and downregulation of iodide-handling genes, whereas *RAS*-like cancers tend to preserve differentiation and iodine avidity (88). *TERT* mutations (alone or in combination with *BRAF* or *RAS*) are associated with RAI refractoriness (89–91). Transcriptomic classifiers have the potential to more accurately predict treatment sensitivity than mutational data alone. In particular, loss of thyroid-specific gene expression (e.g., SLC5A5, TG, TPO) and enrichment of MAPK and PI3K-AKT signaling pathways are hallmarks of poor RAI response (26, 66).

For advanced or RAI-refractory thyroid cancer, next-generation sequencing remains essential to identify actionable alterations. Up to 87% of patients harbor targetable mutations or fusions, and in 57% an FDA-approved therapy is available, most commonly involving *BRAF^V600^*^E^ or *RET* fusions (92). Targeted kinase inhibitors, including *BRAF*/MEK, *RET*, and *NTRK* inhibitors, as well as redifferentiation and combination approaches, have also expanded the therapeutic landscape (93–95). Further studies are required to understand the role of transcriptomic biomarkers in treatment selection. However, potentially, a (transcriptomic) biomarker that reflects sensitivity to RAI could be used to monitor the efficacy of redifferentiation therapy.

## Knowledge gaps and future directions

Although transcriptomic profiling provides biologically rich information, several limitations must be acknowledged. Some challenges are shared with genomic assays, including tumor heterogeneity, variable sample integrity, and the financial costs associated with next-generation sequencing (96). Other limitations are more specific to RNA-based approaches. RNA is less stable than DNA; it is subject to varying degrees of degradation and fragmentation before and after isolation, depending on sample storage temperature, storage time, and preservation medium (e.g., whether the tumor was snap-frozen and stored in liquid nitrogen or formalin-fixed paraffin-embedded) (97). Transcriptomic assays also generate high-dimensional data that require specialized bioinformatic processing and statistical modeling to ensure accurate quantification and interpretation. The lack of standardized procedures for transcriptomic testing compounds the complexity of transcriptomic data. Even in a centralized laboratory environment, implementing transcriptomic testing requires substantial technical expertise, rigorous assay validation, and robust quality-control processes, which may limit scalability and slow clinical adoption.

Despite these limitations, transcriptome-based classifiers have the potential to shift the entire paradigm of PTC management. This shift is overdue, as most other areas of oncology have already embraced transcriptomic profiling to guide treatment pathways. Examples of this include the OncotypeDx Breast Recurrence Score® and Mammaprint. However, multi-centre randomized controlled trials and real-world data will be required before there is broad adoption of these technologies in clinical practice. Future prospective trials should examine the clinical utility of molecular-guided treatment with reference to quantifiable outcomes including recurrence, quality of life, healthcare utilization, and cost-effectiveness.

Currently, there is at least one active prospective trial investigating the clinical utility of a molecular classifier in the postoperative phase to determine need for completion thyroidectomy and adjuvant RAI. Studies investigating the clinical utility of molecular classifiers in the preoperative phase are also planned and should yield valuable insights; guiding the extent of surgery required (i.e. active surveillance vs. hemi-thyroidectomy vs. total thyroidectomy) and potentially even selecting patients suitable for ablative approaches. Other gaps in our understanding of the molecular profiling and behaviour of PTC include the profiling of multifocal disease (do all multi-focal tumour have the same characteristics)?, profiling of micropapillary disease (is this the same disease as conventional PTC)?, and profiling of primary tumours compared to their metastases (do metastases present different profiles requiring a separate approach)?.

Besides prognostication, there are other exciting potential applications of transcriptome-based classifiers. There may be theranostic applications, where targeted molecular guided therapies are guided by molecular subtype; this has become common practice in some cancer types (e.g. hormone receptor and HER2 positive tumours in breast cancer). In the context of PTC, there are opportunities to use molecular classifiers to predict RAI sensitivity and refractoriness, redifferentiation strategies, tyrosine kinase inhibitors (general vs specific), and immunotherapy. In all, there is substantial potential for transcriptomic classifiers to integrate into and improve clinical guidelines such as ATA and NCCN guidelines.

## Conclusion

The integration of transcriptomic insights into the management of PTC promises to refine risk stratification, personalize therapy, and reduce unnecessary interventions. Realizing this potential will require harmonizing molecular data with clinical algorithms through multicenter validation and prospective trials.
